# Concurrent measurement of microbiome and allergens in the air of bedrooms of allergy disease patients in the Chicago area

**DOI:** 10.1186/s40168-019-0695-5

**Published:** 2019-06-03

**Authors:** Miles Richardson, Neil Gottel, Jack A. Gilbert, Julian Gordon, Prasanthi Gandhi, Rachel Reboulet, Jarrad T. Hampton-Marcell

**Affiliations:** 1 Department of Systems Biology, Columbia University, New York, NY 10032 USA; 2 Integrated Program in Cellular, Molecular, and Biomedical Studies, Columbia University, New York, NY 10032 USA; 3 The Microbiome Center, Department of Surgery, University of Chicago, Chicago, IL 60637 USA; 4 Scripps Institution of Oceanography, University of California San Diego, La Jolla, CA 92093 USA; 5 Department of Pediatrics, University of California San Diego, La Jolla, CA 92093 USA; 6 BioScience Division, Argonne National Laboratory, Lemont, IL 60439 USA; 7 Inspirotec Inc, 332 S. Michigan Avenue, Suite 10 32 #1248, Chicago, IL 60604 USA; 8 Department of Biological Sciences, University of Illinois at Chicago, Chicago, IL 60607 USA

## Abstract

The particulate and biological components of indoor air have a substantial impact on human health, especially immune respiratory conditions such as asthma. To better explore the relationship between allergens, the microbial community, and the indoor living environment, we sampled the bedrooms of 65 homes in the Chicago area using 23the patient-friendly Inspirotec electrokinetic air sampling device, which collects airborne particles for characterization of both allergens and microbial DNA. The sampling device captured sufficient microbial material to enable 16S rRNA amplicon sequencing data to be generated for every sample in the study. Neither the presence of HEPA filters nor the height at which the air sampling device was placed had any influence on the microbial community profile. A core microbiota of 31 OTUs was present in more than three quarters of the samples, comprising around 45% of the relative sequence counts in each bedroom. The most abundant single organisms were *Staphylococcus*, with other core taxa both human and outdoor-associated. Bacterial alpha diversity was significantly increased in bedrooms that reported having open windows, those with flowering plants in the vicinity, and those in homes occupied by dogs. *Porphyromonas*, *Moraxella*, *Sutterella*, and *Clostridium*, along with family *Neisseraceae*, were significantly enriched in homes with dogs; interestingly, cats did not show a significant impact on microbial diversity or relative abundance. While dog allergen load was significantly correlated with bacterial alpha diversity, the taxa that significantly correlated with allergen burden did not exclusively overlap with those enriched in homes with dogs. *Alternaria* allergen load was positively correlated with bacterial alpha diversity, while *Aspergillus* allergen load was negatively correlated. The *Alternaria* allergen load was also significantly correlated with open windows. Microbial communities were significantly differentiated between rural, suburban, and urban homes and houses that were physically closer to each other maintained significantly more similar microbiota. We have demonstrated that it is possible to determine significant associations between allergen burden and the microbiota in air from the same sample and that these associations relate to the characteristics of the home and neighborhoods.

**Electronic supplementary material:**

The online version of this article (10.1186/s40168-019-0695-5) contains supplementary material, which is available to authorized users.

## Introduction

The exposome comprises the totality of environmental exposures experienced from conception onwards during a human life and has been associated with human health outcomes [1, 2]. Both allergens and microbes are often associated with inhalable airborne particles; these particles have a substantial impact on human immune response and health outcomes [3]. The indoor air microbiome, or aerobiome, represents an exchange nexus between a number of different sources of bacteria, fungi, and viruses, including humans [4–6], pets [7, 8], and outside air [5]. In the developed world, the average person spends upwards of 87% of their time indoors, including one third of their lives sleeping, during which they inhale significant quantities of indoor air [9]. The human microbiome can dramatically shape the indoor environment through the dispersal of skin and respiratory-associated microbes [4, 6], with approximately 37 million bacterial genomic units and 7 million fungal genomic units released from the average person per hour [10].

Allergens are defined as antigens, including microbial cells and metabolic products, that can lead to a type I immune reaction in people with atopy (asthma, rhinitis, eczema), mainly through the immunoglobulin E (IgE) response pathway. Indoor air quality and antigenic burden are of special concern for human health, with indoor mold exposure shown to correlate with allergic diseases [11]. Conversely, children who are exposed to a greater degree of dust-associated microbial diversity often have lower rates of asthma [12]. Household aerosols can modulate immune response in a protective manner depending on the constituents [13].

Indoor and outdoor air have a large overlap in bacterial composition [5], with indoor air closely resembling outdoor air. Outdoor air has been shown to be a significant contributor to the indoor aerobiome, with 50% or more of the community composition attributable to outdoor sources [14, 15]. However, there is a significant enrichment of human-associated bacteria in indoors relative to outdoor air, and this can vary based on building design [5]. Outdoor air is often significantly more microbiologically diverse than indoor air [5, 14, 16–19]. Sources of bacteria associated with airborne dust indoors can have originated from soil and plant leaf surfaces, and the types and sources can vary by season due to changing ecological conditions, as well as by geographic location [16]. Seasonal variation in the microbial constituents of outdoor air has been demonstrated in Chicago (IL, USA) [20], where a large proportion of the summer aerobiome comprises soil- and leaf-associated bacteria [20]. How this outdoor air variability influences indoor air is highly dependent upon building design [18]. At the same time, the indoor aerobiome is often less diverse than the outdoors and can maintain a greater proportion of bacteria closely related to known pathogens. In outdoor air, these bacteria are often significantly less abundant or below the level of detection [18].

Therefore, indoor airborne allergen exposure comprises both endogenous and exogenous sources [21]. Prior studies have examined the dust and constituent particles in the air of many built environments [22], which have been documented in recent publications from the NHANES 2005–2006 program [23, 24]. Airborne allergen quantification and characterization have, in the past, been more technically challenging than collection of settled dust with a vacuum cleaner. Settled dust is not necessarily representative of inhaled air, and here, we leveraged the Inspirotec electrokinetic air sampling device that allows for the collection of airborne allergens. This device is sufficiently simple to operate that samples can be collected by the patients themselves, in their own homes.

In this study, we deployed the Inspirotec sampling device in 65 Chicago area homes, which were occupied by patients with clinically diagnosed allergy and asthma, as part of a larger study incorporating measurement of common household allergen profiles [25]. The microbiota and airborne allergens were analyzed from the same samples, and differences in allergen and aerobiome profiles between bedrooms were assessed along with survey data from participants, providing a Microbiome Wide Association Study (MWAS) between the different environments.

## Materials and methods

### Sampling design

Six participating Chicago area physicians provided patients with verbal instructions describing the study and Inspirotec sampler usage. Patients were also provided with sampler instruction sheets, HIPAA release forms, a package containing an Inspirotec sampler enclosed in a ziplock bag, and a digital temperature-humidity meter (LaCrosse Instruments, LaCrosse, WI). Labels, on the ziplock bag, were to be completed by patients. They were instructed to run the samplers for 5 days in the bedroom and to note the following information: start time and date, stop time and date, height above floor, distance from bed, temperature and humidity at start, and temperature and humidity at finish. Patients were instructed to plug the sampler in for 5 days, in the bedroom. At the end of the run, they replaced the sampler in the bag and returned it to the physician. Sampling occurred between September and November 2015. A further questionnaire was provided to be completed as a hard copy or as an online version. The project was approved by Quantum Review IRB file #30772. Out of the 102 homes tested, sufficient sample was collected from 86 homes, and of those, 65 had complete metadata from the patient-completed questionnaires and labels. No other special selection criteria were applied. Descriptions of the patients are provided in Gordon et al. [25]. Cartridges were removed from the samplers, and stainless steel electrode strips were released and transferred to 15 ml centrifuge tubes. One milliliter of PBS with 0.02% Tween 20 was added to the tubes and vortexed intermittently over 10 min. Samples were removed from tubes and centrifuged at 15,000*g* for 30 min. The supernatants were removed and subject to immunoassays for the specified allergens. Pellet fractions were resuspended in 100 μl of DEPC-treated water, and 50 μl was transferred to a 0.5-ml tube for DNA extraction and further processing.

### Immunoassay

Samples were assayed for 12 common household allergens by MARIA® (Indoor Biotechnologies) kits comprising three dust mite antigens (Der p 1, Der f 1, Mite Group 2), cat (Fel d 1), dog (Can f 1), mouse (Mus m 1), rat (Rat n 1), cockroach (Bla g 2), molds (Alt a 1, Asp f 1), and pollens (Bet v 1 and Phl p 5). Samples were analyzed with the Bioplex 200 (BioRad) at the University of Illinois, Chicago, Flow Cytometry Facility. Analytical LLODs for MARIA® assays were determined according to the package inserts. Concentrations were presented as pg/m^3^ of air and were based on concentrations determined, sampler flow rates, and flow times. Detailed results of the allergen assays are published elsewhere [25].

### DNA extraction, library preparation, and sequencing

Amplification of the V4 region of the 16S rRNA gene was accomplished using the Earth Microbiome Project 16S protocol [26] adapted for Illumina MiSeq. The 515F-806R region was targeted by region-specific primers that included Illumina adaptors and barcodes. Amplified DNA was sequenced according to Walters et al. [27] on Illumina Miseq. Paired-end reads were demultiplexed with QIIME [28] and merged with VSEARCH [29]. They were then processed with the USEARCH [30] workflow. Sequences were filtered to a maximum expected error of 0.5, dereplicated, and OTU clustered at 97% identity, yielding 4949 OTUs. Taxonomy was then assigned using UCLUST and the Greengenes reference database as implemented in QIIME. To generate a phylogenetic tree, sequences were aligned against the Greengenes reference database version 13.5, and a tree was then generated using fastree. For quality filtering of remaining reads, OTUs not contained in at least 5% of samples were removed, as were OTUs in unidentified phyla. Chloroplast and mitochondrial DNA were removed, and samples were rarefied to 2500 counts per sample. Amplicon sequence data analysis was performed in R, with extensive use of the *phyloseq* [31], *vegan* [32], *DESeq2* [33], and *ggplot2* [34] packages.

### Data availability

Data is available from Qiita, study number 12285, and is available from EBI, accession number PRJEB32320/ERP114984 (Sample Accession Numbers ERS3383113–ERS3383177).

## Results

The homes tested in this study were based on patient participation from allergy clinics [22]. The IRB-approved questionnaire completed by patients can be found in reference [22]. The allergen profiles for these homes were used as the basis for determining possible relationships between aeroallergens and the airborne microbiota.

### Airborne bacterial and archaeal community structure and composition are stable across Chicago area homes

A total of 1144 OTUs were identified from 162,500 sequences across 65 samples after processing. The vast majority, 73.6%, were annotated to 5 dominant phyla. The composition of the aerobiome at this taxonomic level was dominated by the same phyla that predominated Chicago outdoor air in a previous study [20], including *Actinobacteria*, *Bacteroidetes*, *Firmicutes*, and *Proteobacteria*. The most abundant OTU was annotated as *Staphylococcus*, a genus commonly associated with the human respiratory tract [7] and skin [35]. The most abundant OTUs in the dataset were also the most prevalent, defined as the number of distinct samples an OTU was identified in, suggesting that each bedroom is dominated by similar, common, and highly abundant organisms. This relationship held across all OTUs, where OTUs with a larger number of reads were also present in more samples than less abundance OTUs (Spearman’s correlation, *r* = 0.85; *p* < 0.001).

A core microbiota of 31 OTUs was present in more than three quarters of the samples, comprising around 45% of the relative sequence counts in each bedroom, and their distribution is even and widespread (Additional file 2: Figure S1). While human body-associated bacteria, such as *Corynebacterium*, *Staphylococcus*, and *Enterobacteriaceae*, were highly abundant, bacteria commonly associated with the outdoor environments and outdoor air [16] were also present, including *Sphingomonas* (leaf, water, and soil associated), which have been previously shown to comprise a significant proportion of outdoor air microbiota in Chicago during the summer [20].

### Both physical and socioeconomic factors significantly impact diversity

The room condition that most significantly contributed to the variance in observed bacterial alpha diversity was the self-reported presence of open windows (Spearman’s correlation, *p* < 0.001, correlation 0.419). Open windows have been found to increase the diversity of the indoor air microbial community [18]. However, while the diversity of microbes increased, the composition was not significantly altered (Bray-Curtis Mantel, *p* = 0.482), suggesting that opening windows increases the number of rare organisms characterized by the amplicon sequencing approach. The presence of flowering plants in the vicinity was also significantly correlated with increased alpha diversity (Wilcoxon signed rank sum test, *p* = 0.042). Income was positively associated with diversity (Spearman’s correlation, *r* = 0.390; *p* < 0.001), but not with differences in community composition (Bray-Curtis dissimilarity, Mantel *p* = .203).

Interestingly, the presence or absence of HEPA filters did not significantly impact the observed bacterial community. This may be because the HEPA filter does not selectively remove specific taxa, and therefore, while it may reduce overall bacterial biomass, it does not influence diversity or structure. There has always been an open question as to whether the compartment of aerosol closer to the floor, which may represent a larger size population closer to collected settled dust, would compositionally differ in response to the height above the floor, since a different size fraction may be sampled. The height of sampling did not significantly impact the composition or diversity of the community (Additional file 1: Table S1). There was also no correlation between any allergen and height above the floor [22]. Further, there was no relationship between temperature, humidity, or distance from the bed with changes in the microbial community. (Additional file 1: Table S1).

As observed in prior studies, dogs significantly increased the alpha diversity of the bacterial community (Wilcoxon signed rank test, *p* = 0.034). A subset of OTUs associated with *Porphyromonas*, *Moraxella*, *Sutterella*, and *Clostridium*, along with family *Neisseraceae*, was significantly enriched in homes with dogs, relative to that of homes without dogs (Additional file 3: Table S2). No bacteria were significantly enriched in homes with cats when compared to controls.

### Allergen loads of fungal and canine origin interact with community diversity

**Fig. 1:**
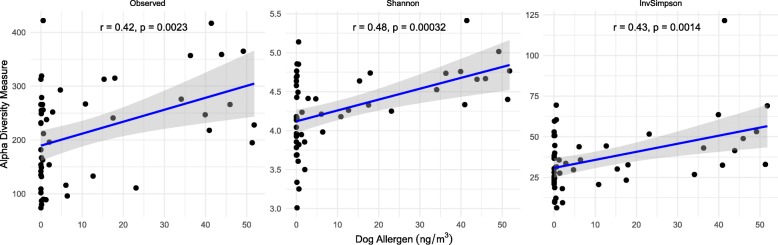
Alpha diversity measures (Shannon index, inverse Simpson index, and the observed number of OTUs) are shown for dog allergen. Linear regressions were generated for each measure alongside 95% shaded confidence intervals and the Spearman correlation statistics

**Fig. 2:**
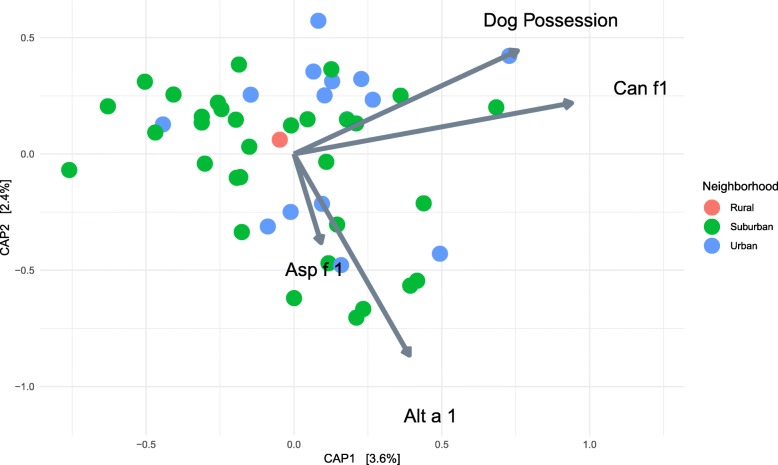
Canonical analysis of principal coordinate (CAP) analysis including both dog ownership and the number of dogs. The axes created by a combination of dog allergen, dog presence, and both mold allergen levels are plotted against each other. The arrows indicate the change in the microbial community in response to an increase in the variable associated with the arrow across the CAP axes

**Fig. 3:**
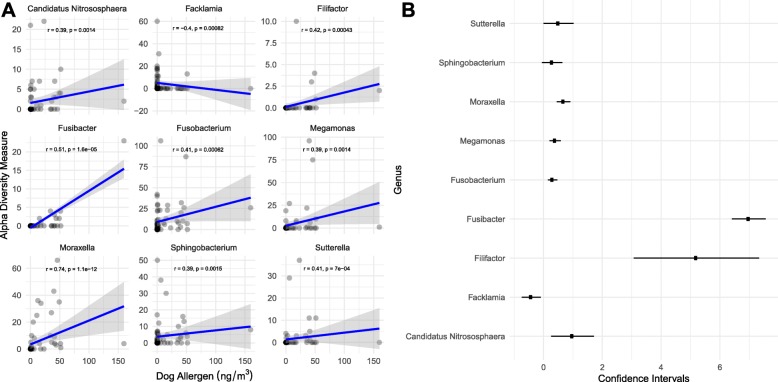
**a** Linear regressions for significant (*p* < 0.01) Spearman’s rank correlations in response to dog allergens were plotted with shaded 95% confidence intervals. Spearman’s correlation statistics were plotted per group. **b** Effect sizes with standard error were plotted for each significant genus

Dog allergen levels significantly positively correlated with bacterial alpha diversity (Table 1), and linear regressions generated from Spearman’s rank correlations for alpha diversity metrics against dog allergen loads demonstrate a significant positive association (Fig. 1). However, the associated correlation between bacterial beta diversity, dog possession, and dog allergen burden shows slightly differential associations (Fig. 2), which could be indicative of different dog breeds altering dog allergen burden or a dose-dependent response associated with the number of dogs that occupy the home. By contrast, both mold allergens had different magnitudes of effect, but similar directionality. The relative abundance of bacterial taxa such as *Aliivibrio* and *Friedmanniella* was most significantly positively correlated with dog allergen load, but both taxa had relative abundances < 1%. *Moraxella*—previously demonstrated to be differentially abundant between the presence and absence of dogs—was also significantly enriched with greater dog allergen load (Fig. 3). At the same time, previously identified members of the genus *Porphyromonas*, *Sutterella*, and *Clostridium* were not significantly associated with allergen loads. Importantly, the taxa that were significantly associated with dog allergen load only slightly overlap with those that were significantly enriched in homes with dogs, again suggesting some differentiation between the presence of dogs and dog allergen burden.

**Table 1:** Correlating allergen load with the bacterial Shannon diversity metric

Allergen (pg/m^3^)	Spearman’s correlation			Mantel test	
	*t* value	*p* value	Correlation	Mantel *R*	Significance
*Alternaria* (Alt a 1)	2.089	0.040*	0.233	− 0.087	0.777
*Aspergillus* (Asp f 1)	− 2.502	0.015*	− 0.276	− 0.003	0.394
Birch (Bet v 1)	1.578	0.119	0.178	− 0.068	0.771
Cat (Fel d 1)	1.128	0.263	0.128	− 0.113	0.889
Dog (Can f 1)	3.279	0.002**	0.35	0.055	0.22
Mouse (Mus m 1)	0.317	0.752	0.036	− 0.004	0.372
Roach (Bla g 2)	0.458	0.648	0.052	0.038	0.252
Timothy grass (Phl p 5)	1.626	0.108	0.183	− 0.074	0.756
Total dust mites (Der f 1 + Der p 1 + MG2)	0.996	0.323	0.113	− 0.138	0.923

* means *p* < 0.05, ** means *p* < 0.01

Specific mold allergens also significantly correlated with bacterial alpha diversity (Table 1). *Alternaria* allergen load was positively correlated, while *Aspergillus* allergen load was negatively correlated. The *Alternaria* allergen load was also significantly correlated with open windows (Spearman’s correlation, *p* < 0.05, correlation 0.355), potentially confounding its relationship with diversity.

### Influence of geography on the microbiota and allergen load

It is possible that the presence of a “core microbiome” between homes in this study may have resulted from a shared air environment; therefore, we explored how the outdoor air environment impacted the indoor air bacterial community. Urban and suburban communities may harbor different outdoor bacteria, reflecting differences in land use, population density, etc. We found that urban and suburban bedrooms were not significantly different in composition (ANOSIM, Bray-Curtis dissimilarity, *R* = − 0.077 *p* = 0.844) nor in diversity (AOV, *F* = 0.844, *p* = 0.435), indicating that the category of neighborhood as defined in this study does not directly influence the aerobiome. Zip code did not have an effect of community composition (Bray-Curtis dissimilarity, Mantel *R* = 0.005, *p* = 0.475) but did have an effect on diversity (AOV *F* = 4.04, *p* < 0.001).

**Fig. 4:**
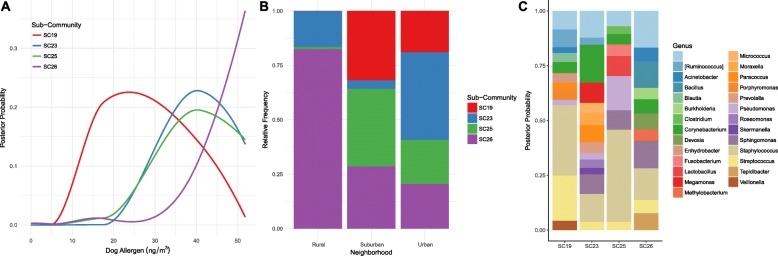
**a** Posterior probabilities of significant (noted by 95% credible intervals) microbial sub-communities (19, 23, 25, and 26) were fitted against dog allergens using a local regression. **b** The relative frequency of the significant sub-communities was observed for samples grouped by neighborhood—urban, suburban, and rural. **c** The posterior probability of frequently co-occurring taxa found within each sub-community was observed at the genus level

We further explored how neighborhood interacted with dog allergen levels, as dogs are known as a source of outdoor bacteria [8]. Dog allergen concentration was significantly positively correlated with microbial diversity, suggesting dogs act as a source of bacteria not represented in the aerobiome. To determine the association between dog allergens and the surrounding neighborhood, we employed Bayesian topic models to partition microbes into sub-communities based on their co-occurrence in samples. Then, we tested the prevalence of each sub-community in relation to dog allergen and how those sub-communities related to neighborhood type. Based on 95% credible intervals, a Bayesian version of confidence intervals, four sub-communities had a significant interaction with dog allergen load. Sub-communities 19, 23, and 25 showed normal distributions in relation to allergen load, with 23 and 25 skewed towards increased probability at higher loads; meanwhile, sub-community 26 showed an exponential increase with greater allergen load (Fig. 4a). The relative frequency of each sub-community in rural, suburban, and urban homes suggested differential abundance with rural homes dominated by sub-community 26 and urban homes showing more equal distribution of the different sub-communities (Fig. 4b). The probability distribution and thus composition of microbial genera differed significantly between each sub-community, with sub-community 26 showing an increased relative proportion of *Acinetobacter*, *Bacillus*, and *Tepidibacter* (Fig. 4c). Linear regressions showed *Staphylococcus* was the only differentially abundant taxa across all sub-communities (*p* < 0.01, 95% CI [0.49, 0.98]).

**Fig. 5:**
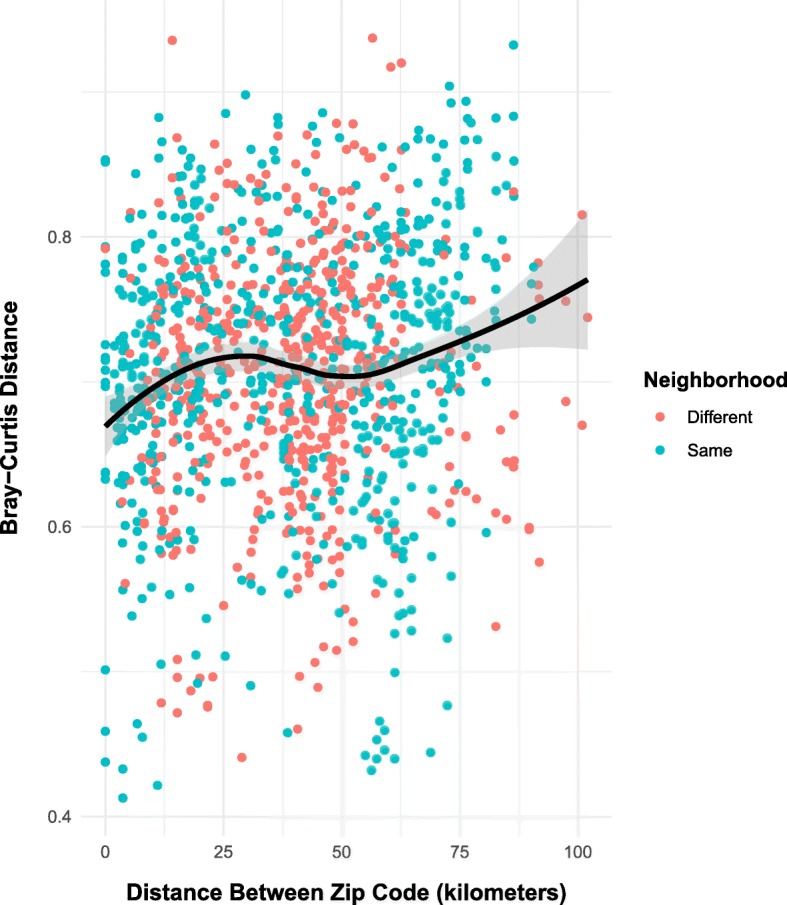
The relationship between geographic distance and bacterial community distance. The distance between the zip code of each home was calculated and labeled as being from the same neighborhood (urban or rural) or a different neighborhood type

Finally, we examined if geographic proximity between homes was associated with microbial community similarity. Physical proximity may capture similar outdoor air environments between adjacent homes, which may not be captured by neighborhood, which stretches over a wider geographic area. Bray-Curtis dissimilarity (beta diversity) was significantly correlated with the geographic distance (Mantel *r* = 0.100, *p* = 0.048), indicating that homes that are physically close have significantly more similar microbial communities, where the distance has its greatest effect at less than 25 km (Fig. 5). Thus, physical proximity significantly changes the similarity of the aerobiome, while neighborhood classification does not appear to have a significant effect.

## Discussion

In this study, we characterize the various factors that influence the diversity and composition of the aerobiome of bedrooms in the Chicago area. A small number of prevalent bacteria form a “core microbiome,” suggesting either a common source of these bacteria or common factors that promote a similar community. If there is a significant outdoor component, the core microbiome would represent a group of ubiquitously distributed organisms. By contrast, if homes independently assemble, the presence of common core organisms could represent similar conditions across homes selecting for similar communities. There is some evidence that indoor air communities assemble in a manner independent of the specific occupants of the respective built environment, suggesting a large outdoor component [14]. Further, it is unclear if these core organisms represent a set of unique strain-level organisms or an ensemble of closely related organisms. As our sequences are clustered into operational taxonomic units at 97% nucleotide identity, our data is not sufficient to distinguish between closely related organisms. Further, this may not be improved by sub-OTU methods, as 16S data is often not capable of distinguishing strain-level organisms [36]. Finally, geographic proximity is significantly positively correlated with bacterial community similarity, which supports an outdoor contribution, as homes that are close together would have more similar input from outdoor air.

Despite the large number of allergens sampled, few appear to have a relationship to the aerobiome, which may be due to insufficient sample size or lack of microbial association. This may be addressed by a more focused study with a large sample size for many of the allergens. At the same time, a number of allergens appear to have links to changes in community composition. Correlation of dog ownership with diversity and changes in the composition of the microbial community is well known [7, 8], and thus, the observation that both dog allergen and dog ownership appear to have this effect is unsurprising. The difference between microbial communities that associate with dog ownership and dog allergen load may be explained by the fact that these are not identical populations. At one end of the distribution, homes with dog ownership and no dog allergen detected could indicate a tight home where dogs are excluded from the bedroom and the air supply to the bedroom is well controlled. At the other end, homes with dog allergen and no dog ownership could represent a sub-population in which dog allergen is introduced by some outside traffic entering homes from unknown external sources, but not with an attendant-associated microbial community. This study demonstrates that dog allergen load as well as geographical location can influence the aerobiome captured in homes and that distinct microbial sub-communities arise in relation to these factors. Interestingly, this relationship was not observed for cats, which could be because cats are commonly indoor-only pets and likely would not contribute to the dispersal of exogenous microbiota into residential homes [8].

Mold allergens also had a significant correlation with bacterial diversity. Their presence may not represent a causal relationship, but may be the result of a possible unidentified common causal factor. *Alternaria* is a genus of saprophytic fungi found in soil and decaying plant matter [37] and is a common allergen in humid regions [38]. It has also been found to be a constituent of indoor air, especially in households with indoor plants [39]. *Aspergillus* has both outdoor and indoor sources [40–42], including the air and water systems, and is closely associated with indoor fungal particle emissions, while *Alternaria* is much less so [43]. This suggests that while *Aspergillus* is an integral member indoor aerobiome [44], *Alternaria* presence may be correlated with an increased outdoor contribution to indoor. We found that *Alternaria* was significantly correlated with open windows, suggesting that the association with increased bacterial diversity may be due to increased outdoor air contribution and not due to a co-association with *Alternaria* spores. Despite the quantification of these two fungal allergens, there is a huge amount of fungal diversity that was not sampled in this study. The direct measurement of fungal sequences through ITS sequences was not collected due to budgetary constraints, but is not only technically feasible but would be an excellent supplement to this data. This would allow for the examination of fungal diversity as it relates to the sampling factors presented in this study.

Besides allergens, factors that could affect sampling, such as the distance from the bed of a participant, humidity, height above the floor, and temperature, did not impact the bacterial community recovered from participant bedrooms. Interestingly, humidity does, in contrast, affect the load of a number of different allergens [25]. The fact that height above the floor does not impact microbial community confirms that there is enough mixing and averaging of the air properties over the 5-day sampling. In earlier studies, we have reported no significant variation in bacterial population diversity or abundance compared with filter sampling as a reference method [45]. Therefore, there is no bias introduced by the fact that the capture efficiency measured with latex particles was 23% [25]. Further, the 1-μm aerodynamic diameter chosen in this standardized reference method is aimed at being equivalent to *Bacillus anthracis* and surrogate *Bacillus* species [45, 46], demonstrating that our method is capable of capturing the microbial community.

In contrast to data presented here, O’Connor et al. [47], who used vacuum cleaner dust collection for analysis of allergens and the microbiome, were able to obtain sufficient material for microbiome profiling in only 56% of their samples. By contrast, in this study with Inspirotec sampling of the air, we have collected sufficient material for 100% of the samples. In their population, they observed correlations between allergens from cockroach, mouse, cat, and dog and bacterial taxa that were significantly different between homes of asthmatic and non-asthmatic children. The population here was not selected by disease state, but all bedrooms were used by people with an allergy or asthma diagnosis. There were no significant differences in our dataset for allergens or microbiome based on self-reported symptoms, which likely is due to a lack of statistical power. We are not aware of any other study comparing allergen and microbiome content in the same samples.

The inter-relation between the inhaled air of the aerobiome and the human airway microbiome and relation to human health is not well-understood. However, the method described here allows for a more thorough interrogation, due to its ability to recover the 1.2 μm and below particle size fraction [45]. This fraction enters human airways, including particle sizes that are capable of deep penetration in the airways and triggering of asthma [25]. This is likely to be more informative than measurements of vacuumed dust or settled dust. Additionally, the ease of deployment and running of the devices by the patients themselves, such as in this study, shows that it lends itself logistically to large-scale deployment and citizen-based science projects. This will be facilitated by cost savings through future manufacturing scale-up. Thus, we have demonstrated a robust, effective, and easy to use air sampling device that allows for a better understanding of the aerobiome.

## Additional files

Additional file 1: **Table S1.** Community diversity based on selected abiotic variables. (CSV 343 b)

Additional file 2: **Figure S1.** The relative proportion of OTUs at the order level that are most prevalent, designated as contained in more than 75% of samples. Each sample is scaled to relative abundance. (PNG 967 kb)

Additional file 3: **Table S2.** Differentially Abundance OTUs between households with and without dogs. (CSV 56 kb)
