# Supplementary figures and images for: Concurrent measurement of microbiome and allergens in the air of bedrooms of allergy disease patients in the Chicago area
Source: Microbiome. 2019 Jun 3;7:82. doi: 10.1186/s40168-019-0695-5 (PMC6547563; doi:10.1186/s40168-019-0695-5)

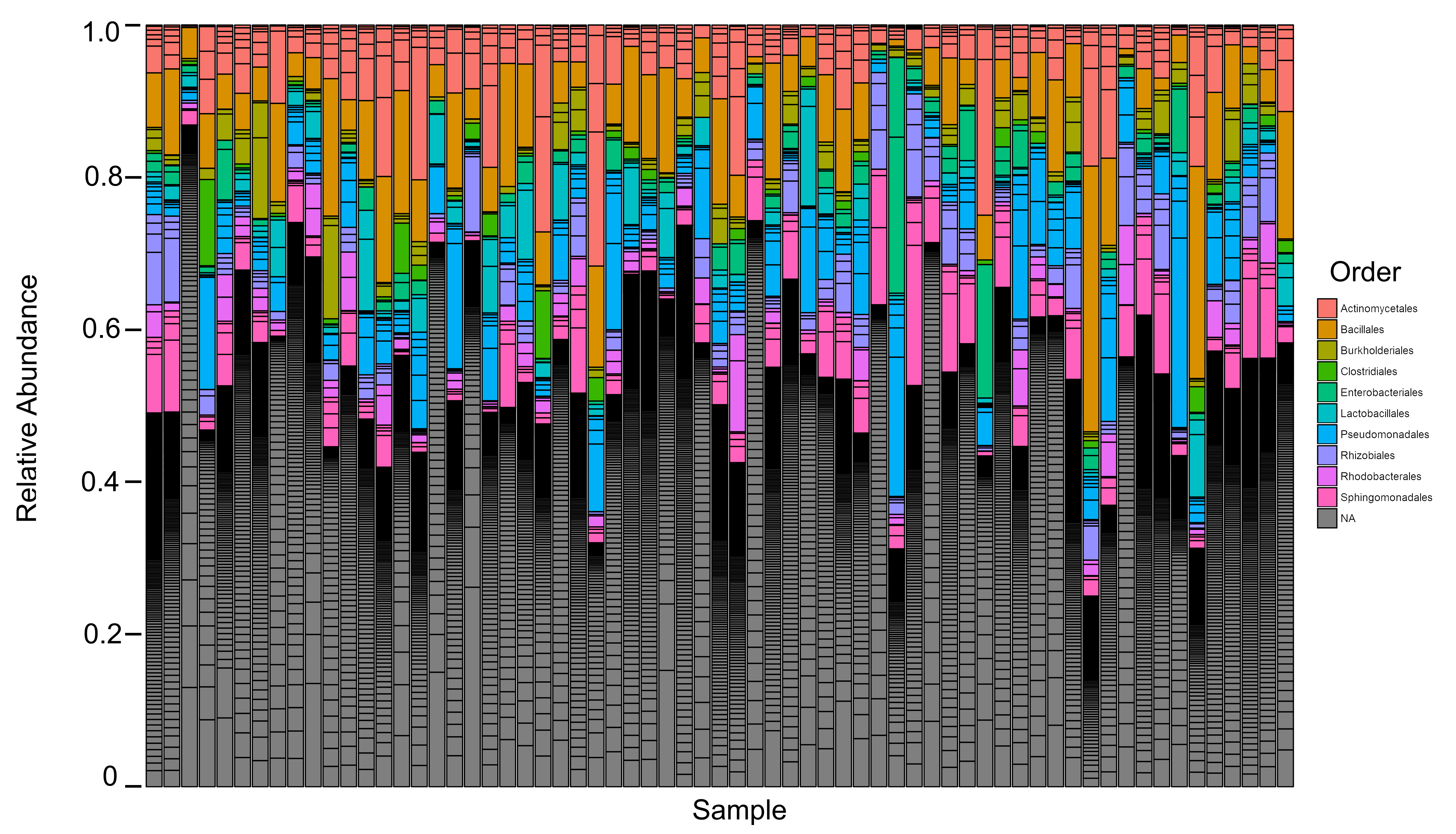

Supplement: Supplementary file 2 — Figure S1. The relative proportion of OTUs at the order level that are most prevalent, designated as contained in more than 75% of samples. Each sample is scaled to relative abundance. (PNG 967 kb) [file 40168_2019_695_MOESM2_ESM.png]
